# Small Angle Neutron Scattering at the National Institute of Standards and Technology

**DOI:** 10.6028/jres.098.003

**Published:** 1993

**Authors:** B. Hammouda, S. Krueger, C. J. Glinka

**Affiliations:** National Institute of Standards and Technology, Gaithersburg, MD 20899

**Keywords:** ceramic morphology, macro-molecules in solution, metal alloys, microstructure, polymer structure, protein-DNA complexes, sintering, small-angle neutron scattering

## Abstract

The small angle neutron scattering technique is a valuable method for the characterization of morphology of various materials. It can probe inhomogeneities in the sample (whether occurring naturally or introduced through isotopic substitution) at a length scale from the atomic size (nanometers) to the macroscopic (micrometers) size. This work provides an overview of the small angle neutron scattering facilities at the National Institute of Standards and Technology and a review of the technique as it has been applied to polymer systems, biological macromolecules, ceramic, and metallic materials. Specific examples have been included.

## 1. Introduction

Small angle neutron scattering (SANS) instruments are very useful for the investigation of microstructures of 1 to nearly 500 nm in size in various materials such as porous media, polymers, ceramics, metals, etc. The very low energies of thermal neutrons make them an excellent non-destructive probe of microstructure since neutrons are very penetrating in most materials. The ability to substitute deuterium (D) for hydrogen (H) in macromolecular complexes makes SANS a unique technique for probing macromolecular conformations in synthetic and biological polymers.

Examples of research areas well-suited to the SANS technique include the determination of void sizes and their distributions in porous media such as metals, coals, shales, gels, etc. as well as the investigation of particle agglomeration in unsintered ceramic bodies and evolution of pore populations during sintering. SANS is also useful for the investigation of the morphology of polymer (“plastic”) materials and of the relationship between their structures and properties. Structural changes of synthetic and biological macromolecules in solution with changing environment can be monitored and the internal structure of aggregated systems such as membranes, micelles (water-oil-soap), microphase segregated copolymers, etc. can be determined. SANS can also provide structural information that is helpful in the understanding of the thermodynamics of two-phase systems such as polymer blends, metal alloys, composites, etc.

Since neutrons have a magnetic moment, the magnetic scattering from magnetic materials can also be studied. SANS is useful for the determination of the range and degree of order in magnetic structures and for the measurement of critical properties of magnetic systems.

The National Institute of Standards and Technology has been operating an 8 m SANS instrument since the early 1980s. By the summer of 1992, two 30 m high resolution SANS instruments were fully operational. This work describes the three SANS instruments at NIST and gives an overview of SANS research opportunities in the areas of polymer science, biology, ceramics, and metals.

## 2. The NIST SANS Instruments

The 8 m NIST SANS instrument benefited from the installation of a cold source at the NIST reactor in 1987, which resulted in substantial increases in intensity for that instrument. After the construction of a guide hall, completed in 1990 as part of the Cold Neutron Research Facility (CNRF), the 8 m SANS instrument was moved to the end of one of the newly installed neutron guides. The first 30 m SANS instrument (the Exxon/Univ. of Minn. SANS), also installed in the guide hall, began operation in the Spring of 1991 while the second 30 m instrument (the CHRNS-SANS) began operation in the summer of 1992.

### 2.1 The 8 m SANS Instrument

The 8 m SANS spectrometer [1] is a general purpose instrument with a broad range of accessible scattering vectors (i.e., scale lengths probed), continuously variable wavelength and a number of possible sample environments. The instrument characteristics are listed in Table 1. The instrument components include a helical velocity selector with 25% wavelength resolution (which means high intensity on sample), a 4.5 m evacuated flight tube preceding the sample and a 3.5 m scattering flight path that can be rotated about the sample position to reach larger scattering angles. A computer-controlled sample chamber that houses up to six positions is available for measurements at near ambient temperatures. The sample chamber can be removed to accommodate bulkier sample environments such as a cryostat or displex. Neutrons are detected with an area detector with an active area of 64 × 64 cm. The low-*Q* limit on the instrument is achieved with multibeam converging pinhole collimation.

### 2.2 The Two 30 m SANS Instruments

**Fig. 1:**
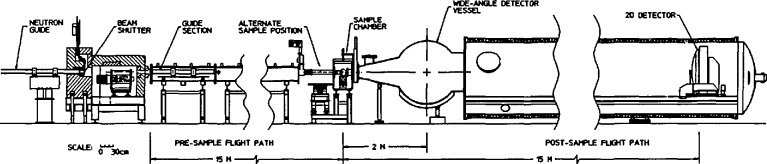
Schematic drawing of the NIST/EXXON/Univ. of Minn. 30 m SANS instrument.

**Fig. 2:**
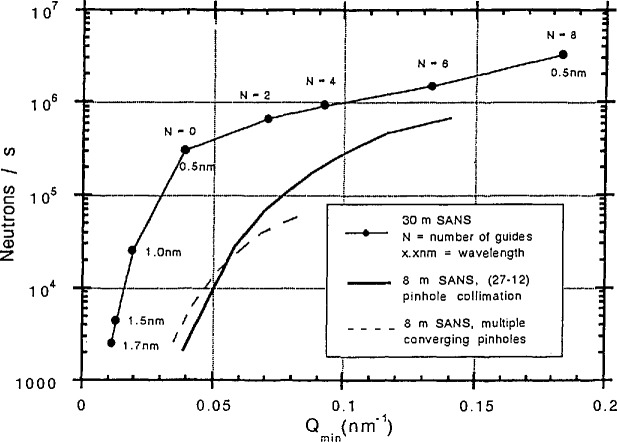
Intensity at the sample position for the NIST/EXXON/Univ. of Minn. 30 m SANS instrument and for the 8 m SANS instrument.

The two 30 m SANS spectrometers are high resolution, wide scattering vector range instruments that combine and improve upon some of the best features of existing long flight path instruments in the world. The characteristics for the two instruments are listed in Table 2 and a schematic of the NIST/EXXON/Univ. of Minn. 30 m SANS is shown in Fig. 1. The neutron intensity at the sample position of that instrument (for a 1.5 cm diameter sample) is shown in Fig. 2 which also shows the intensity at the sample position for the 8 m SANS instrument.

The design characteristics of these two 30 m SANS instruments have many similar features including a computer-controlled multidisk velocity selector, an adjustable incident flight path with neutron guide sections that can be moved into the beam in order to bring the effective neutron source closer to the sample therefore enhancing the intensity, and a beam polarizing device (supermirror transmission polarizer) to be developed. The scattering flight path can be varied between 2 and 16 m by changing the detector position. Both instruments provide two different sample positions. The first position features a multi-position sample chamber which can accommodate up to nine sample positions. Upstream, the second sample position consists of an adjustable sample table for bulky sample environments. A number of sample environments are available for the SANS users. These include heating blocks and circulating baths for measurements at temperatures ranging from −20 to 200 °C, a closed cycle He^3^ cryostat for measurements in the 30 mK to 300 K range and furnaces for measurements at temperatures up to 2000 °C. Shearing cells are available for measuring liquids and gels under shear. In addition, measurements can be made in magnetic fields up to 7 T.

A set of user friendly software packages for data acquisition, reduction and analysis as well as instrument control have been written. A microvax 4000^1^ computer is used for data acquisition and reduction while Macintosh computers are used for on-line color imaging of the data. The ability to take reduced data away on IBM PC or Macintosh floppy disks is also provided.

## 3. Small Angle Neutron Scattering from Polymers

### 3.1 Introduction

Small angle neutron scattering is a unique diagnostic technique to probe the morphology of polymer materials. Due to the ability to replace hydrogen atoms by deuterium, this technique can monitor specific macromolecular conformations in polymer solutions as well as in bulk polymer systems. It can also monitor concentration fluctuations in phase decomposing binary mixtures such as homopolymer and/or copolymer blends. Contrast variation methods (mixing deuterated and non-deuterated polymers or solvents) are used to change the “color” of polymer chains making them more or less visible to neutrons similarly to the staining method in electron microscopy.

Due to the fact that SANS spectra are not abundant in prominent features (as for example diffraction or NMR spectra are), the SANS technique relies heavily on modeling of the scattering intensity. Macromolecular systems can be modelled fairly well owing to the pioneering work of many scientists such as P. Flory (Gaussian chains, theta temperature, etc.), H. Kuhn (polymer chain stiffness, etc.), W. Stockmayer (gelation, etc.), B. Zimm (dilute solutions, etc.), P.G. de Gennes (random phase approximation, scaling ideas, etc.) to name only a few. The SANS technique has shown with no ambiguity, for instance, that polymer coils follow random walk trajectories when they are in the bulk state [2–4]. This means that correlations between monomers along the chain backbone are screened by other surrounding chains, so that the chain “forgets” quickly (after one or two steps) about where its other parts are. This screening is less effective in polymer solutions and self avoiding walk statistics are more appropriate making coils more swollen or more collapsed depending on whether the constraints on the walk are repulsive or attractive (i.e., depending on the nature of the monomer-solvent interactions). Flory introduced a characteristic transition temperature (called the theta temperature) for which the monomer-solvent interactions are equivalent making the chain appear “ideal” (as if it were in a bulk environment). Below this temperature, the solvent cannot dissolve the polymer, while above it, it becomes a good solvent.

Blending of polymers is necessary for better controlled physical properties of polymeric materials. Unfortunately, most polymer alloys are incompatible (i.e., most polymers do not like to mix). The binary mixtures that are known to mix have been very precious systems for studying the thermodynamics of phase separation. For example, polystyrene and polyvinylmethylether (PS/PVME) show [5] a lower critical solution temperature (LCST) since they are miscible at room temperature but phase separate with a spinodal decomposition temperature around 140 °C when the fractions are about 30/70 and molecular weights 435,000/188,000. The mixing of low molecular weight polystyrene and polybutadiene (PS/PB), on the other hand, has an upper critical solution temperature (UCST) phase diagram [6] and shows mixing upon heating from ambient temperature (spinodal temperature is around 37 °C for a mixture of 77/23 dPS/hPB with *M*_w_=900/4,500). Conformations in the miscible region, concentration fluctuations close to the immiscible region as well as the delimitation of the spinodal line have been well understood for a number of polymer blend systems [5–14] using the SANS technique with deuterium labeling of one of the components.

A method of forcing different polymers to mix is to chemically copolymerize them at the small block level. Most copolymers can therefore mix at the macroscopic level but show microphase separation. Here also, the SANS technique supplemented with specific deuteration of one of the blocks has been valuable [15–18] in mapping out chain conformations in each of the various possible morphologies (lamellar, rod-like, spherical).

In cases where detailed models of polymer systems are not available, generic SANS methods such as Guinier plots are used to extract characteristic sizes. Other direct methods based on linear fits of combinations if *I*(*Q*) and *Q* vs powers of *Q* are used to extract scaling laws which are signatures of various kinds of morphologies. *I*(*Q*) is the measured scattered intensity and *Q* is the scattering wavenumber. For instance a slope of − 2 in a plot log[*I*((*Q*)] vs log(*Q*) hints that chains are in ideal (Gaussian) conformations.

### 3.2 SANS from Polymer Solutions

Polymer solutions constitute a bench-mark for understanding basic interactions between monomers and solvent molecules. Effects of temperature, concentration and chain stiffness have been thoroughly investigated [19–23] using the SANS technique.

Temperature effects are investigated using Flory’s swollen chain approach. Variation of the radius of gyration, *R*_g_, of a single chain (i.e., its effective statistical size) with the number of monomers *N* in the chain (representative of the molecular weight *M*_w_) follows the simple mean field scaling law: *R*_g_*=N^ν^a*^2^/6, where *a* is the monomer size and *ν* is the Flory excluded volume parameter (*ν* = 1/2 for ideal chains, *ν* = 1/3 for collapsed chains and *ν* = 0.6 for fully swollen chains). Of course scattering data contains contributions from inter-chain contributions which are accounted for through concentration effects on the scattered intensity *I*(*Q*) or on the structure factor *S*(*Q*). These two quantities are related to each other by the coherent scattering cross section {*b*}^2^, monomer volume fraction *ϕ*_P_, and machine constant *C*:

$$I\left(Q\right)=C{\left\{b\right\}}^{2}\phi_{P}S\left(Q\right).$$ (1)

In dilute solutions, only binary chain interactions contribute to the interaction energy, making the second virial coefficient the dominant term in a virial expansion. Such interactions occur mainly through single contacts (as pointed out by Zimm [24]) making the static structure factor, *S*(*Q*), a simple two-term expansion in terms of the monomer volume fraction:

$$S\left(Q\right)=S_{0}\left(Q\right)\left[1-\nu \phi_{P}S_{0}\left(Q\right)\right].$$ (2)

Here, *S*_0_(*Q*) is the single-chain structure factor and *ν* is the excluded volume during the interaction between monomers and solvent molecules which is related to the monomer-solvent Flory interaction parameter *K* as:

$$\nu =\left(1/\phi_{s}-2K\right).$$ (3)

Moreover, *ϕ*_s_ = 1 − *ϕ*_P_ is the solvent volume fraction. The reciprocal form of Zimm’s expansion can be derived directly [25,26] using the random phase approximation (RPA) and was a major tool (Zimm plot) for extracting single chain properties from dilute polymer solutions. This method of obtaining single chain properties is hampered by low scattering signals due to the low polymer concentrations involved. Another method allowing the use of concentrated polymer solutions was introduced in 1980 [27,28].

The high concentration method of extracting single chain properties such as the radius of gyration, the monomer size or the persistence length (which is a measure of chain stiffness) consists in using mixtures of deuterated and non-deuterated polymers of the same molecular weight in semidilute or concentrated solutions. The single chain structure factor can be obtained from the scattering data by varying the fraction of deuterated chains while keeping the total polymer concentration constant.

**Fig. 3:**
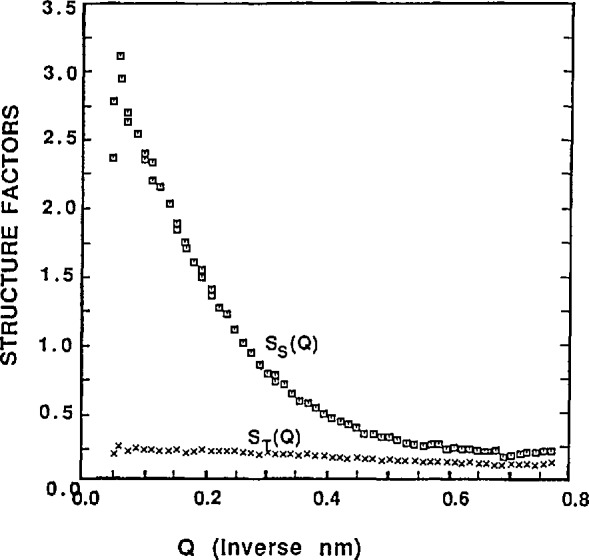
Single-chain and total structure faetors for a concentrated solution of mixtures of deuterated and nondeuterated polystyrene (dPS, hPS) in toluene. The total polymer concentration was 22% and the relative deuterated fractions were varied from 0% to 85%.

A representative example [29] of this method is included here. Figure 3 shows the single-chain and the total scattering structure factors for dPS in hPS of *M*_W_ = 65,000 as shown in Ref. 29. More than two (for redundancy) relative concentrations (*ϕ*_dps_/ϕ_hps_ = 20%,40%,60%,85%) were measured in toluene solutions with fixed concentration (ϕ_P_ = 22%). The total scattering structure factor is seen to be flat (as expected) owing to the fact that all monomers contribute to that term equally.

The use of high concentrations increases the signal-to-noise ratio making it much easier to monitor single chain properties. This high concentration method made Zimm plots less preferable in SANS data analysis.

### 3.3 SANS from Blends of Homopolymers

**Fig. 4:**
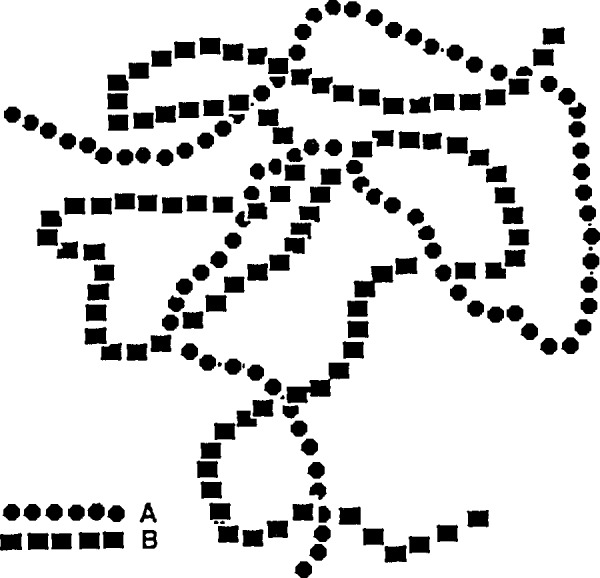
Schematic representation of a two-component polymer blend. Only two maeromolecules are represented for simplicity.

In the case where polymers are homogeneously mixed (blended), the RPA is also very useful. A two-component blend (called A and B for convenience) and shown in Fig. 4 can be looked at as a polymer component (say A) in solution in the other component (B). The partial structure factors, such as S_AA_(*Q*), can be related to the bare structure factors *S*_0AA_(*Q*) and *S*_0BB_(*Q*) (when no interactions are present) for AA and BB correlations as:

$$\begin{array}{r} 1/\phi_{A}S_{\mathrm{AA}}\left(Q\right)=\left[1/\phi_{A}S_{0\mathrm{AA}}\left(Q\right)\right]\\ +\left[1/\phi_{A}S_{0\mathrm{BB}}\left(Q\right)\right]-2K_{\mathrm{AB}} \end{array}$$ (4)

where *ϕ*_A_ and ϕ_B_ are the partial volume fractions (ϕ_A_ + ϕ_B_ = 1). The bare structure factors are usually replaced by a Debye function (Gaussian coils) which is a good representation of ideal non-interacting chains.

This RPA result for the structure factors is an extension [30,31] of Eq. (2) in which the solvent volume fraction ϕ_s_ is replaced by ϕ_B_*S*_0BB_(*Q*). This result was first introduced by de Gennes [25] and is applicable to situations where a mean field approach is valid such as in dilute solutions, concentrated solutions, melts and blends. It does not give good results in semidilute solutions where chains start overlapping (multiple contacts are not accounted for in the RPA) but do not overlap enough to begin screening intrachain correlations.

The scattered intensity for an incompressible blend is given by:

$$I\left(Q\right)=C{\left\{b_{A}-b_{B}\right\}}^{2}S_{\mathrm{AA}}\left(Q\right),$$ (5)

where *b*_A_ and *b*_B_ are the scattering lengths of monomers *A* and *B*, respectively. A correlation length *ξ* can be extracted from a fit of the data (taken at different sample temperatures *T*) to the Ornstein-Zernike form:

$$I\left(Q\right)=C{\left\{b_{A}-b_{B}\right\}}^{2}N_{A}\phi_{A}/\left(1+Q^{2}\xi^{2}\right)$$ (6)

in the Guinier region. Plots of *I*^−1^(*Q*) vs *T*^−1^ or of the extracted *ξ*^2^ vs *T*^−l^ show a linear behavior which deviates sharply close to the spinodal temperature (where concentration fluctuations start becoming large, signaling the onset of phase separation). Extrapolation of this linear behavior gives the spinodal temperature *T_s_* (for which both *I*(*Q*) and ξ blow up). This method, when repeated for different volume fractions, has allowed a precise mapping of the spinodal line for many compatible polymer blend mixtures.

**Fig. 5:**
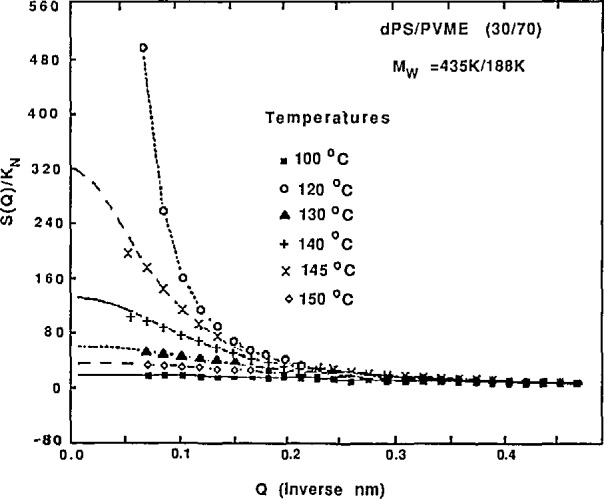
Corrected SANS intensity for a blend of 30/70 dPS/PVME taken at various temperatures from the one-phase region to the two-phase region.

**Fig. 6:**
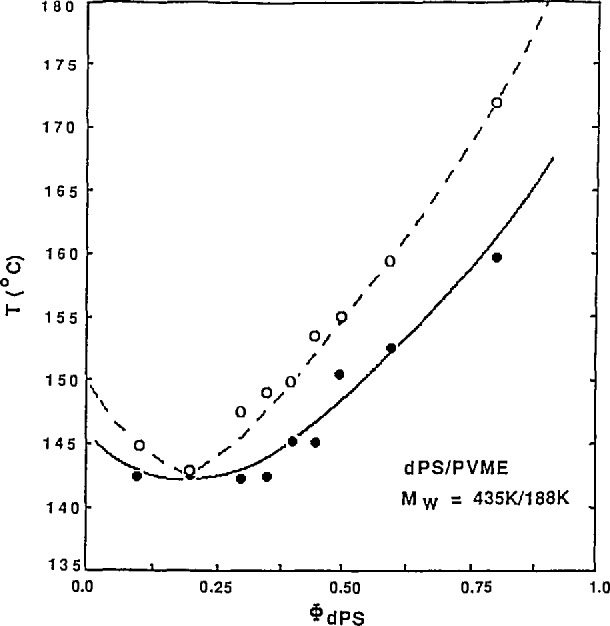
Phase decomposition diagram as obtained from SANS measurements for the 30/70 dPS/PVME blend system.

As an example, scattering SANS data and the resulting phase diagram [5] for a dPS/PVME blend are shown in Figs. 5 and 6. The molecular weight were *M*_w_ = 435,000 and 188,000 and the volume fractions were 30/70 for the two components (dPS and PVME), respectively. The Flory interaction parameter *K*_dPS/PVME_ was found to vary inversely with temperature away from the spinodal temperature as the mean field theory predicts.

### 3.4 SANS from Copolymers and Copolymer/Homopolymer Mixtures

The RPA approach for a blend of two homopolymers can be extended to include an arbitrary number of homopolymers and/or copolymers using a matrix notation formalism:

$$X^{-1}\left(Q\right)=X_{0}^{-1}\left(Q\right)+\nu$$ (7)

where the matrices *X* and *X*_0_ represent the interacting and bare structure factors respectively and the matrix *v* contains the various excluded volumes *v*_AA,_ *v*_BB_ and *v*_AB_ The various structure factors for the interacting system are obtained from the inverse of Eq. (7). In the case of block copolymers, the off-diagonal elements of the bare structure factors matrix *X*_0_(*Q*) are non-zero and represent cross correlations between different blocks. These results were derived by Benoit and coworkers [31]. Sakurai et al. [32] used a similar approach to sort out the effects of microstructure and deuterium labeling in random copolymers of 3,4 polyisoprene (hPI) with 1,4 hPI in 1,2 dPB with 1,4 dPB (copolymer A-B in copolymer C-D). The effective Flory interaction parameter *K*_eff_ shows an LCST phase separation behavior while the individual interaction parameters *K*_AB_ *K*_AC_ etc., all clearly show a UCST behavior. The overall behavior results from an interplay among all of these partial interaction parameters.

### 3.5 Other SANS Applications from Polymers

The SANS technique has been very successfully used in many other polymer problems. For example, SANS has been instrumental in the understanding of polymer conformations in semicrystalline polymers. For instance, it was found that the radius of gyration of deuterated chains in crystalline polyethylene is comparable to that in the disordered phase [33–38] (before crystallization). This points to the fact that only local conformational changes are required during crystallization. SANS has also found extensive applications in understanding polymer adsorption on micron size latex particles [39–43], in charged polymer systems [44–45], and in gels and interpenetrating networks [46–54], etc.

One of the goals of materials science is to seek structure/property relationships. SANS has contributed [55–58] to materials science by providing conformational changes when polymer materials have been subject to various treatments. For instance, the degree of anisotropy of polymer chains has been investigated in partially deuterated (5%) polystyrene (*M*_w_ = 250,000) samples that were hot stretched above the glass-rubber transition temperature (110 °C) and then quenched back to ambient temperature. It was found [55–56] that macro-molecules follow the external (mechanical) stretching affinely up to elongations that double the sample length. Beyond that elongation, plastic deformations take over and the chains start slipping past each other in order to release the externally applied stress. Similar methods were also used [57–58] to monitor macromolecular orientation along a shear band that developed after notching and compressing partially deuterated PS samples.

### 3.6 Future Research Horizons

Since its introduction in the early 1970s, the SANS technique has found a great deal of applications in the field of polymer research covering applications all the way from cutting edge science to applied routine characterization. Newly introduced methods (such as the high concentration method or the random phase approximation) have brought renewed interest in the SANS technique making it an ever growing research tool for polymer scientists. A recent literature search of the Chemical Abstracts database based on the two keywords “neutron” and “polymer” came up with 480 articles that were published between 1980 and 1990, 424 of which used the SANS technique.

The two 30 m SANS instruments that have been constructed at NIST will certainly help alleviate such an unsatiable demand for beam time. These two long flight path instruments have resolutions from the near atomic (1 nm) to the near micrometer (500 nm) length scales. This, combined with good intensity on sample, is opening up a wide range of experiments in the area of polymers. One such experiment will consist in the investigation of shear induced phase separation of polymer solutions. Solutions of high molecular weight (*M*_w_ = 10^6^) dPS in DOP solvent show a UCST phase diagram with a spinodal temperature around 13 °C. They are also known to phase separate at room temperature when under high shear rates. Monitoring of the chain conformations will yield a better understanding of the thermodynamic phase diagram and of its shifting under shear. Because of the large *R*_g_’s involved, such an experiment requires the use of a low-*Q* instrument such as a 30 m instrument.

Another experiment will use a time slicing feature that is being included in the data acquisition software of the 30 m SANS instruments at NIST. This feature allows the recording of sequential time spectra with small time mesh increments. A dPS/hPB blend is in the two-phase region at room temperature and goes to the one-phase region either by heating over 40 °C or by shearing the mixture. We intend to investigate the conformational changes that are associated with the return to the two-phase region after quenching back down to room temperature or after cessation of the shear flow.

## 4. Small Angle Neutron Scattering from Biological Macromolecules

### 4.1 Introduction

SANS is an important complement to techniques such as electron microscopy, hydrodynamic measurements and biochemical assays in the study of biological structures since macromolecules are measured in solution without further preparation. The main advantage of neutrons for the study of biological macromolecules is that the lighter elements such as carbon, hydrogen, nitrogen, and oxygen all have similar neutron scattering lengths. In addition, the large difference in the scattering lengths of hydrogen and deuterium makes it possible to study different components of a macro-molecular complex *in situ* by substituting D_2_O for H_2_O in the solution and/or D for H in the complex. This makes SANS useful for the study of two-component systems such as protein-nucleic acid or lipid-protein complexes and even more complex systems such as cells and vesicles. If deuterium labelling is used, individual subunits can be located within multi-subunit proteins. In addition, conditions can be manipulated to highlight interactions between macromolecule and solvent. Several reviews on the applications of SANS have been published in the last several years [59–62].

### 4.2 Method

A macromolecule will only scatter neutrons when its scattering length density, *ρ*, is different from that of the surrounding solution. Since SANS is a low resolution technique, the solvent is assumed to be infinite and homogeneous, with a scattering length density, *ρ*_s_. The difference between the scattering length density of the macromolecule and that of the solvent, Δ*ρ* = *ρ* − *ρ*_s_, is known as the contrast. The intensity of the scattered neutrons, can be written as [63]

$$I\left(Q\right)=n{\left(\Delta \rho \right)}^{2}<{\left|\int_{v}\exp\left(iQ\cdot r\right)d^{3}r\right|}^{2}>,$$ (8)

where *n =N/V* is the number of macromolecules per unit volume and < > denotes an average over all possible orientations of the macromolecule. *Q* is the momentum transfer of the neutron, with a magnitude of

Q=4π(sinθ)/λ, (9)

where 20 is the scattering angle. At zero scattering angle,

$$I\left(0\right)=n{\left(\Delta \rho V_{p}\right)}^{2},$$ (10)

where *V*_P_ is the particle volume which is inaccessible to the solvent [60].

**Fig. 7:**
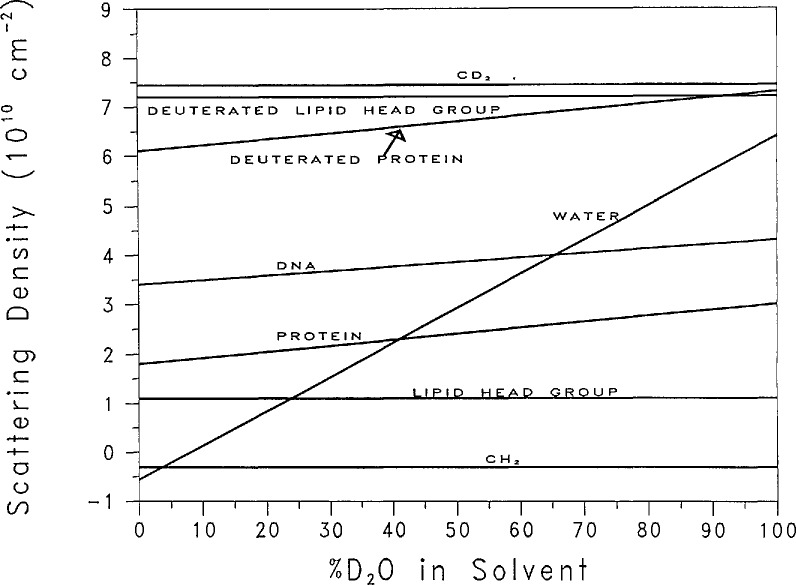
Scattering density vs %D_2_O in the solvent for various biological components.

By manipulating the contrast by changing either *ρ* or *ρ*_s_, the intensity of the scattered neutrons at *Q* = 0 can be increased or decreased, depending upon the value of Δ*_ρ_*. If *ρ*_s_ is changed by adjusting the ratio of H_2_O to D_2_O in the solvent, then Δ*ρ* varies linearly with the concentration of D_2_O (%D_2_O) in the solvent [60]. Thus, a plot of $\sqrt{I\left(0\right)}$ vs. %D_2_O yields a straight line which crosses the *x*-axis at the point where the neutron intensity vanishes. This is the match point of the molecule. This match point is especially useful for composite systems, such as protein-DNA or lipid-protein complexes. Using the method of “contrast variation” [64], the scattering from one component can be minimized by adjusting the H_2_O/D_2_O ratio in the solvent, thus allowing the scattering from the other component to dominate the total scattering. Figure 7 shows the scattering length density from several components of biological complexes as a function of %D_2_O in the solvent. Note that the scattering density from water varies from − 0.562 × 10^10^ cm^−2^ for H_2_O to 6.4 × 10^10^ cm^−2^ for D_2_O.

In the small angle limit, the Guinier approximation for the scattered intensity,

$$I\left(Q\right)\sim I\left(0\right)\exp\left(-Q^{2}R_{g}^{2}/3\right),$$ (11)

where *R*_g_ is the radius of gyration of the macromolecule, applies in the range *QR*_g_ *≤* 1. Both *I*(0) and *R*_g_ may be determined from a plot of ln[*I*(*Q*)] vs *Q*^2^.*I*(0) is related to the molecular weight of the molecule and *R*_g_ to the shape. Beyond the *Q* region where Eq. (11) is valid, *I*(*Q*) must be compared to model curves in order to gain further information about the macromolecular structure. SANS has been useful for rapid measurements of the radius of gyration and molecular weight of macromolecules in solution [65]. Rapid characterization can be provided in solution in a non-”invasive” manner. More extensive studies aiming to further characterize biological macromolecules are described below.

### 4.3 Multi-Component Systems

Systems such as protein-nucleic acid complexes are well suited to SANS studies since there is a large difference in scattering length densities between protein and that of nucleic acid. Thus, the location of the nucleic acid component relative to the protein component can be ascertained. Conformational changes in the protein upon nucleic acid binding can also be quantified. This technique has been applied successfully to a number of systems of which the nucleosome [66], DNA-gyrase [67] and amino-actyl tRNA synthetase systems [68,69] are just a few examples.

The prokaryotic ribosome has been the subject of extensive SANS studies. SANS has been used not only to locate the RNA component in the 30S and 50S ribosomal subunits [70–73], but also to map the location of the constituent proteins with respect to one another using the label triangulation technique [70,74–76]. This method can only be successful if pairs of deuterated proteins are incorporated into the ribosome so that the distance between them could be measured. The shapes of these proteins have also been determined from measurements of isolated proteins in solution [77] and from measurement of the proteins *in situ* using specific deuteration [70]. Deuterium labelling has proven successful for the measurement of distances between subunits in the DNA-dependent RNA polymerase [78–80] and from tryptophan synthase [81].

### 4.4 Membrane Proteins

Membrane proteins are an integral part of biological membranes. The complexity of the membrane composition makes them difficult to study *in situ.* Yet, the hydrophobic nature of membrane proteins also can pose a problem for solution studies. The development of detergents which allow the extraction of active membrane proteins [82] has helped to alleviate this problem. Since a membrane protein is soluble in detergent, its radius of gyration and molecular weight can be obtained using the contrast variation technique. The method was applied successfully to the ATP/ADP transport protein [83].

### 4.5 Cells and Vesicles

**Fig. 8:**
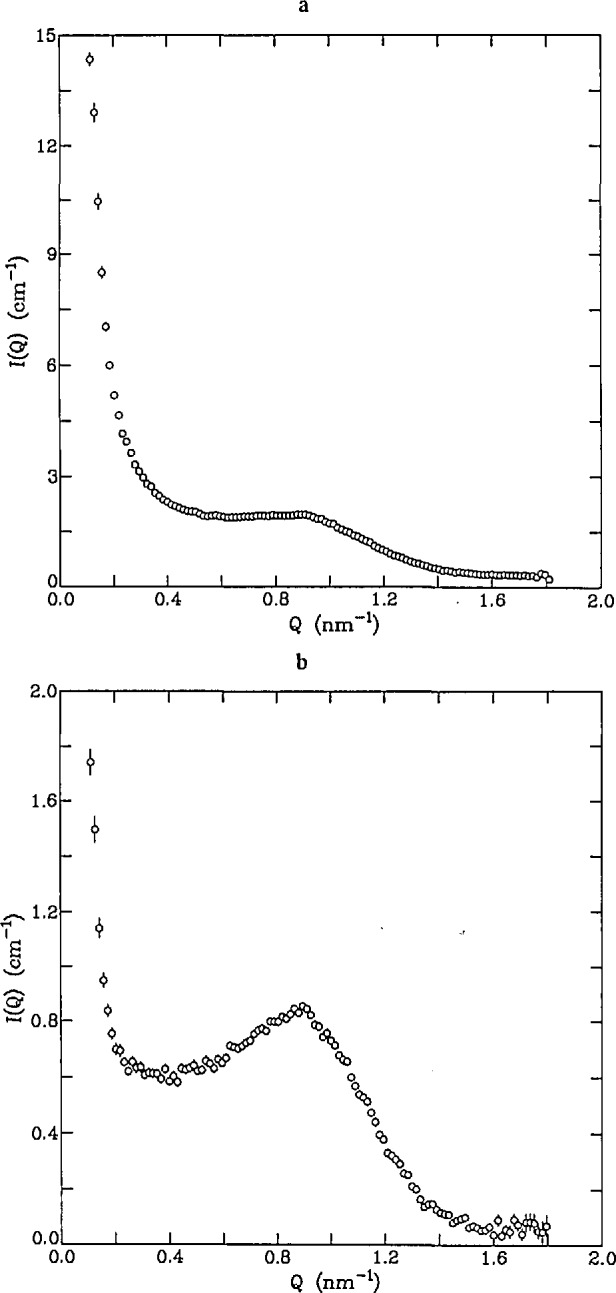
Scattered intensity from blood cells in a) 100% D_2_O and b) H_2_O (0% D_2_O). The scattering a low *Q* values is due to the cell membranes whereas the peak at *Q* = 0.85 nm^−1^ is due to interacting hemoglobin molecules.

The cores of cells and vesicles can be studied *in situ* using the method of contrast variation, provided that the scattering length density of the core constituents is sufficiently different from that of the surrounding membrane. The protein cores of red blood cells [84] and neurosecretory vesicles [85] have been studied by using contrast variation to minimize the scattering from the cell membrane. Figure 8 shows the scattered intensity from red blood cells in 100% D_2_O, where the membrane scattering dominates the total intensity, and in H_2_O, where the protein (hemoglobin) scattering dominates. The sharply-rising portion of the curve at low-*Q* values corresponds to the cell membrane whereas the peak in the intensity at approximately *Q* = 0.85 nm^−1^ is due to interacting hemoglobin molecules. While the overall intensity is smaller in the H_2_O case, the ratio of hemoglobin to membrane intensity is much larger under the same conditions. Similarly, the magnetic scattering from magnetite particles in magnetotactic bacteria was measured in 30% D_2_O, where the scattering from the bacterium was minimized with respect to that of the magnetite particles [86].

### 4.6 Interaction with Solvent

A hydration shell around a macromolecule can be measured directly with SANS if its scattering length density differs from that of the bulk solvent. Using deuterated glycerol and alcohol aqueous solvents, the hydration shell around ribonuclease A was measured [87]. Charged macromolecules can be surrounded by a volume of dense solvent due to the exclusion of salts in their immediate vicinity. In an H_2_O/D_2_O contrast variation experiment, such a region surrounding tRNA was studied under different salt conditions [88].

### 4.7 Future Directions

The development of cold neutron beams has made the SANS technique more useful for the study of biological macromolecules in dilute solutions. However, even at dilute concentrations, purified material is needed in milligram quantities. Improved biochemical techniques for producing molecules and complexes in these quantities will allow many more systems to be examined. Specific deuteration could also be fully exploited if the investment in sample preparation is reduced.

## 5. Small Angle Neutron Scattering from Ceramic Materials

### 5.1 Introduction

The SANS technique is an important tool for measuring residual porosity in sintered ceramics since other techniques such as mercury porosimetry and gas adsorption are not available for measurement when the pores are closed. However, conventional SANS measurements will only detect residual pores smaller than 100 nm. Additionally, many ceramic materials contain microstructural fractures that are larger than 100 nm, including initial porosity, particle agglomeration, and impurity effects in the compacted powder and during the early sintering stages [89]. Finally, conventional SANS cannot be used readily to study the porosity of green or partially-sintered ceramics due to the high porosity of these materials.

The range of sizes applicable to neutron scattering has been extended to 10 μm using multiple small angle scattering (MSANS) techniques [90,91]. The MSANS formalism can be used to measure thicker as well as denser systems in which the coherent elastic neutron scattering cross-section is dominated by multiple scattering. Consequently, powder samples with large particle agglomerates can be measured at densities approaching 50% of theoretical density (TD). In addition, large pores in green compacts (50–60% TD) can be measured with MSANS even though the porosity is very high (40–50%). Together, conventional SANS and MSANS can effectively be used to cover the full range of relevant microstructure sizes in ceramic systems.

Using the MSANS formalism to extend the range of sizes measurable with small angle neutron scattering to between 0.08−10 μm, the evolution of microstructure as a function of thermal processing, which is important for the development of process models in ceramics, has been addressed [92,93]. In addition, the processing/microstructure relationships in ceramic materials as a function of green body density and sintering aids such as MgO [94] have been investigated.

### 5.2 MSANS Theory

Unlike conventional SANS, the scattering in the MSANS regime is dominated by multiple neutron scattering. The radii of the scattering pores, or particle agglomerates, are the same order of magnitude as the mean distance a neutron can travel through the material before being scattered or absorbed. This means that the neutrons scatter from only one pore, or particle agglomerate, at a time even though they may scatter many times before leaving the sample. Thus, there is no contribution to the measured scattering from interference between scatterers.

The interaction of neutrons with matter is characterized by the phase shift *ν* that a plane wave undergoes in traversing a particle of radius *R*. This phase shift determines the shape of the single-particle differential scattering cross section, *dΣ*(***Q***)/*d****Ω***), as a function of the scattering wavevector ***Q***, where |***Q***| = 2π*ϵ*/λ and ϵ< <1is the scattering angle and λ is the neutron wavelength. *ν* depends upon Δ*_ρ_*, the contrast of the particle or void relative to the scattering matrix such that

υ=2ΔρRλ. (12)

In the SANS diffraction regime, *v* < < 1, whereas in the MSANS regime, 0.1 ≤ *ν* ≤ 2.0.

**Fig. 9:**
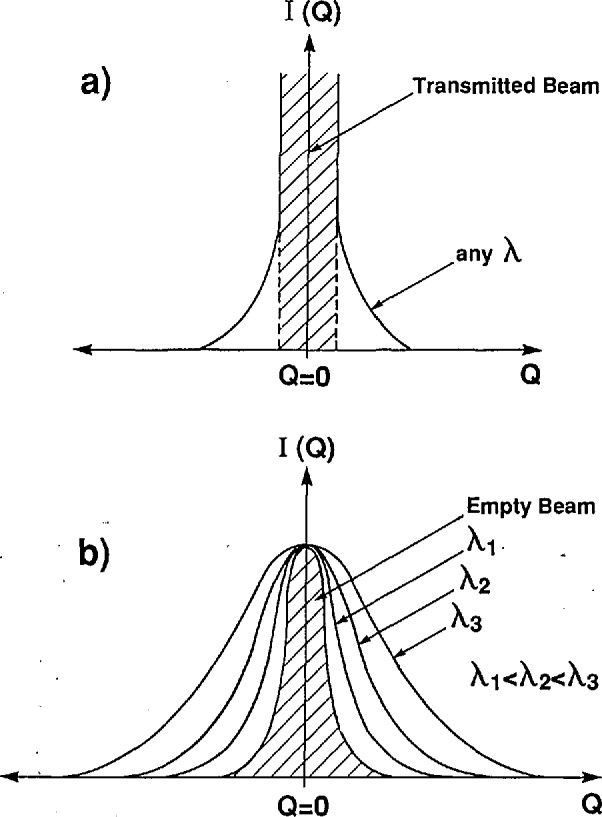
Representation of a typical SANS scattering curve for a) conventional SANS and b) multiple SANS.

In conventional SANS, the scattered intensity as a function of *Q* is independent of neutron wavelength and its shape near *Q* = 0 depends only on the particle dimensions. Figure 9a represents a typical SANS scattering curve. The scattered intensity around *Q* = 0 cannot be measured directly because it lies in the same region as the transmitted beam, which is 10^3^ to 10^7^ times more intense than the scattered beam. Therefore, a beamstop is usually employed to prevent the transmitted beam from reaching, and thus damaging, the neutron detector. For any particle shape, the particle size can be described by its radius of gyration *R*_g_, or Guinier radius [95], which applies at the small *Q* portion of the scattering intensity curve.

In MSANS, the intensity of the transmitted beam is immeasurably small and the width of the scattered intensity curve near *Q* = 0 is broadened far beyond the broadening due to instrumental resolution. The amount of beam broadening is dependent upon the incident neutron wavelength as illustrated in Fig. 9b, where representative MSANS curves are shown for different wavelengths incident on a single sample. The intensities have been normalized such that *I*(0) = 1.0. In each case, the scattered intensities, *I*(*Q*), have a curvature, *r*_c_, near *Q*=0 and can be approximately described as a Gaussian with a width proportional to λ^2^. An effective radius, *R*_eff_(0), where the 0 refers to the small *Q* region near *Q* = 0, can be determined for the scatterers from the wavelength dependence of *r*_c_ using the MSANS formalism represented in Eqs. (2.12–2.15) of Berk and Hardman-Rhyne [90,91]. The MSANS formalism in effect replaces the standard Guinier analysis in the small *Q* region of *I*(*Q*) where *R*_eff_(0), rather than *R*_g_, defines the size of the scatterers. However, both *R_s_* and *R*_eff_(0) are volume-weighted measures of pore radius.

Even under conditions where the low *Q* portion of the scattering curve is dominated by multiple scattering, the large *Q* portion of the scattering curve, where *QR* ≤ 10, follows [90] single particle Porod [95] behavior where the scattered intensity is proportional to *Q^−^*^4^. Thus the standard Porod analysis [95] can be applied to the large *Q* region of *I*(*Q*) independent of the existence of MSANS at low *Q*.

In the Porod region, the scattered intensity can be written as

$$I\left(Q\right)=PQ^{-4}+B,$$ (13)

where *B* is a background term and *P* is Porod’s constant defined as

$$P=2\pi {\left(\Delta \rho \right)}^{2}\left(S/V\right),$$ (14)

where *S/V* is the normalized total surface scattering area per unit volume. Obtained in this manner, *S/V* is independent of the shape of the scattering pores or particle agglomerates. If a spherical shape is assumed, an effective radius, *R*_eff_(∞), where (∞) refers to the large *Q* portion of the scattering curve, can be obtained since *R*_eff_(*∞*) = 3*ϕ*(*V/S*) where ϕ is the volume fraction of scatterers. The number density can be obtained from *N*_P_ = (*S*/*V*)/(4*πR*_eff_(∞))^2^. Unlike *R*_g_ and *R*_eff_(0), *R*_eff_(∞), is a surface area-weighted measure of pore radius.

### 5.3 Creep Cavitation

The failure of ceramics at elevated temperatures often involves the evolution of cavitated grain boundaries. Subsequently, the cavities coalesce to form cracks which then can grow, ultimately causing creep failure. SANS has been used to study the nucleation and growth of creep cavities in sintered alumina [96,97] as well as to characterize the shape of creep cavities in hot-pressed silicon carbide [98,99]. By combining Guinier and Porod results, size distributions for the cavities as a function of creep strain were derived for both materials. SANS was also used to study the materials after post-creep thermal treatment [100] to determine the best way to remove prior creep damage in the form of grain boundary cavitation.

### 5.4 Powders, Compacts, and Sintered Ceramics

The first studies which tested the MSANS formalism involved the characterization of alumina powder [101] and sintered and green compacts of yttrium chromite [102]. The alumina (Al_2_O_3_) powder samples ranged in thickness from 2 to 10 mm and in density from 28% TD to 33% TD. By analyzing the broadening of the scattered intensity curve as a function of neutron wavelength an effective radius of *R*_eff_(0) = 265 nm [101] was determined for the alumina particles. The versatility of MSANS is demonstrated in an experiment [102] in which pressed powder samples of yttrium chromite were measured in the green state (57% TD) and after sintering (94% TD). The green compact showed much more beam broadening than the sintered compact due to the larger pore volume fraction in the green case. However, the fitted *R*_eff_(0) values for both samples were nearly identical, with *R*_eff_(0) = 0.17 μm for the green compact and *R*_eff_(0) = 0.18 μm for the sintered sample.

### 5.5 Pore Evolution and Processing/Microstructure Relationships

**Fig. 10:**
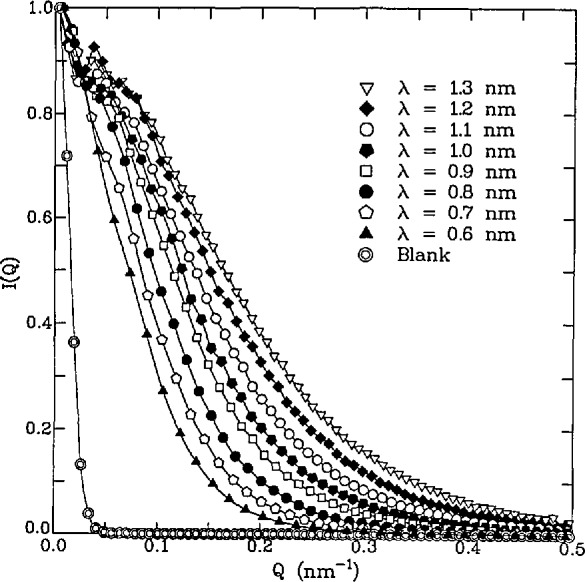
Scattered intensity as a function of neutron wavelength for an 85% TD alumina.

Knowledge of the microstructure evolution as a function of thermal processing is important for the development of process models in ceramics. The pore evolution of crystalline alumina [93] and porous glassy silica [92] have been measured by MSANS as a function of sintering. Figure 10 shows the scattered intensity as a function of wavelength for an 85% TD alumina sample. The beam broadening effect, which is the signature of copious multiple scattering, is easily seen. Using the MSANS formalism, an effective radius, *R*_eff_(0) = 0.18 μm, was determined. Similarly, effective radii were determined for samples ranging in densities from 54% to 97.5% TD for the alumina samples and from 60% to 98% TD for the silica samples. In addition, the effect of green body density and the addition of a sintering aid, MgO, on the pore evolution was examined for the alumina system [94].

**Fig. 11:**
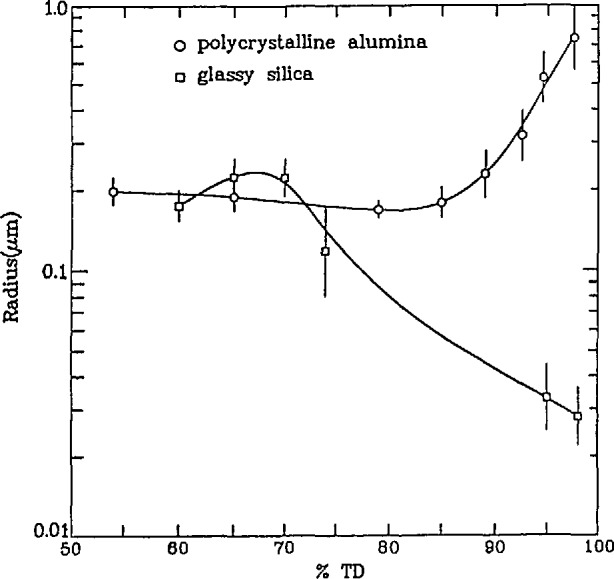
A comparison of the effective pore radius, determined from MSANS measurements, as a function of % TD for a glassy silica system and a polycrystalline alumina system.

By combining the results from the silica and alumina studies, the processing/microstrucrure relationships in both systems were examined in order to gain a quantitative measure of the structural evolution which takes place when different sintering mechanisms dominate [103]. Figure 11 shows the effective pore radius, determined from the MSANS measurements, as a function of %TD for both silica and alumina. Glassy silica sinters by means of viscous flow whereas crystalline alumina sinters by means of surface and volume diffusion. Figure 11 shows the effective pore sizes as a function of density. Clearly, these two major sintering mechanisms lead to very different microstructure evolution signatures.

### 5.6 Future Directions

MSANS measurements typically require on the order of a few minutes to 1 h, which is much more rapid than conventional SANS measurements. If measurements are made at the minimum number of three suitable wavelengths, the time per pore size determination can be quite short. Thus, the MSANS technique makes possible *in situ* studies of the pore evolution in ceramic materials as they are being sintered.

## 6. Small Angle Neutron Scattering from Metallic Materials

### 6.1 Introduction

The same SANS techniques used to study ceramics can be applied to metallic materials as well. These materials also contain microstructure such as precipitates, cavities, phase domains, density fluctuations, magnetic domains, microcracks, and dislocations [104] which can be measured with SANS. The technique is important in developing process models since it provides a quantitative characterization of microstructure.

### 6.2 Precipitate Distribution

**Fig. 12:**
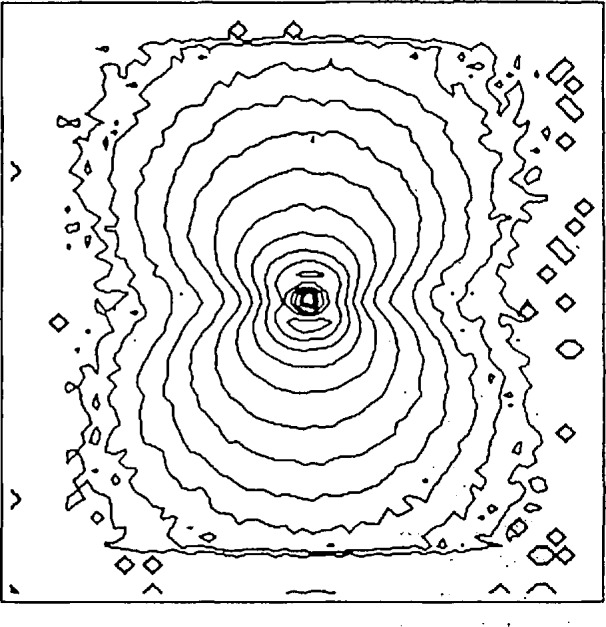
Two-dimensional SANS pattern from an HSLA steel in a horizontal magnetic field.

Precipitates are important in alloys since relatively small changes in precipitate distribution can cause large changes in yield strength or fracture toughness [104]. SANS has been used at NIST to study the precipitate concentration and size distribution in HSLA steel as a function of different heat treatments [105]. HSLA steel is a Cu-Fe system whose strength is developed by the precipitation of a copper-rich phase. Since Fe has a magnetic moment and Cu does not, the scattering contrast between the two components is enhanced, making SANS a unique tool for studying precipitates in this material. Figure 12 shows the 2-D scattering pattern from HSLA steel in a horizontal magnetic field. The magnetic field serves to align the magnetic domains in the Fe matrix to eliminate scattering from domain walls [104]. Thus, the scattering pattern is due to the precipitates and the anisotropy arises because the magnetic scattering is enhanced only in the direction perpendicular to the external magnetic field.

### 6.3 Deformation

SANS has been used to study the effects of plastic deformation on metallic systems. For example, the relationship between microstructure and the permanent volume expansion associated with plastic deformation in maraging steel and aluminum alloys was studied by measuring the scattered intensities before and after straining [104]. Because the intensity increased after straining, the volume expansion could not be caused by an increase of scattering centers such as microcracks or dislocations [104]. Rather, deformation-induced dissolution of precipitates would best explain the SANS results. On the other hand, SANS studies of deformed and annealed Cu and *α*-phase Cu Al single crystals [106] found that the measured intensities resulted from dislocation scattering rather than from bulk segregation of Al in the alloys.

### 6.4 Grain Boundary Cavitation

High temperature failure of alloys is often due to slow nucleation and growth of grain boundary cavities. SANS has been used to study both creep cavitation [107,108] and fatigue-induced cavitation [109,110,111]. In particular, SANS was used to study void growth in alloy 800 during creep [107] by measuring the scattered intensity as a function of creep time for six different samples. More recently, growth rates of grain boundary cavities in Cu during high temperature fatigue [111] have been studied and compared with those resulting from creep. The SANS measurements were combined with precision density measurements to accurately measure cavity surface area and cavity size.

## Figures and Tables

**Table 1:** 8 m SANS instrument characteristics

Source size:	5×5 cm
Wavelength range:	0.48−2.0 nm (velocity selector)
Wavelength resolution:	25% (fixed)
Collimation:	Single pair of circular irises or 7-channel converging beam collimation
*Q*_min_:	0.06 nm^−1^ at 0.6 nm wavelength
	0.035 nm^−1^ at 0.9 nm wavelength
*Q* range:	0.03 to 5.0 nm^−1^
Sample size:	0.4−2.0 cm with pinhole collimation
	1.5 cm with converging collimation
Flux at sample:	10^3^ to 5 × 10^5^ n/cm^2^·s depending on slit size and wavelength.

**Table 2:** 30 m SANS instrument characteristics

Source size:	5×5 cm
Wavelength range:	0.4−2.0 nm (velocity selector)
Wavelength resolution:	7%−30% (continuously tunable)
*Q*-Range:	0.01−6 nm^−1^ (CHRNS-SANS)
	0.01−10 nm^−1^ (NIST/EXXON/Univ. of Minn. SANS)
Sample size:	0.5−2.5 cm
Expected flux at sample:	10^3^ to 10^6^ n/cm^2^·s depending on slit size, wavelength, and source to sample distance

## Footnotes

1 Certain commercial equipment, instruments, or materials are identified in this paper to specify adequately the experimental procedure. Such identification does not imply recommendation or endorsement by the National Institute of Standards and Technology, nor does it imply that the materials or equipment identified are necessarily the best available for the purpose.
